# Breaking the habit: Evidence-based approaches to smoking cessation

**DOI:** 10.6026/973206300220800

**Published:** 2026-02-28

**Authors:** Susmitha Murella, Harsimran Kaur, Vasundhara Gunti, Tvishita Polakala, Radhika Kanani, Gouthami Krishna Juttu

**Affiliations:** 1 Department of Dentistry, NTR University of Health Sciences, Government Dental College and Hospital, Vijayawada, India; 2 Department of Dentistry, Swami Devi Dyal Hospital and Dental College, Haryana, India; 3 Department of Dentistry, SRM Institute of Science and Technology, SRM Kattankulathur Dental College and Hospital, Kattankulathur, Chengalpattu, Tamil Nadu, India; 4 Department of Dentistry, The Oxford Dental College and Hospital, Bangalore, Karnataka, India; 5 Department of Dentistry, K.M.Shah Dental College & Hospital, Gujarat, India; 6 Department of Dentistry, NTR University of Health Sciences, Army College of Dental Sciences and Hospital, Telangana, India

**Keywords:** Tobacco cessation, vaping, social determinants of oral health, oh4l, pharmacotherapy, behavioral interventions, public health

## Abstract

Smoking is a major public health concern that significantly impacts the oral cavity, the primary site of exposure. Tobacco consumption has been linked to conditions such as leukoplakia, oral cancer and impaired wound healing and severe periodontal disease. At the same time, vaping disrupts the oral microbiome and compromises implant stability, both influenced by social, economic, behavioral and political determinants. Evidence suggests that multicomponent interventions, including dental-based programs combined with community initiatives, behavioral strategies such as Motivational Interviewing and the 5Rs framework and pharmacotherapies such as varenicline, bupropion or NRTs are essential for improving cessation outcomes and reducing tobacco-related oral health risks. Thus, we show the consequences of conventional smoking and vaping on oral tissues, periodontal structures, sensory perception, dental implants and saliva.

## Background:

Smoking, including cigarettes, vaping, reverse smoking and other tobacco products, remains a primary global health concern with profound implications for oral health [1]. The oral cavity, as the first point of contact for smoke, is highly vulnerable to damage. Despite growing awareness of smoking's systemic effects, its oral consequences are often under- addressed [2]. Smoking contributes to a wide range of oral diseases, from periodontal disorders to malignancies [3]. It affects nearly every component of the oral cavity, including mucosal surfaces, periodontal structures, implants, salivary composition and sensory perception, ultimately impacting systemic health [4]. Smokers consistently exhibit greater bleeding on probing, deeper periodontal pockets and more pronounced clinical attachment loss than nonsmokers. In addition, cigarette smoking is strongly linked to some of the most fatal oral and respiratory cancers, along with other frequent consequences such as dental staining and various periodontal diseases [1]. Oral health professionals play a vital role in identifying these diseases and raising awareness on prevention; therefore, it is of interest to describe evidence- based approaches for tobacco cessation.

## Oral precancerous and cancerous lesions:

Oral leukoplakia, a white patch that cannot be scraped off or classified as another lesion, is frequently observed in smokers. It may appear smooth or fissured and typically affects the buccal mucosa. Depending on smoking frequency, lesions may be diffuse or localized. Research indicates that 2-6% of leukoplakia cases may progress to malignancy [3]. Smokers exhibit altered pigmentation in the oral mucosa, with RGB photographic evaluations showing darker coloration due to chronic tobacco exposure. Smokers are at increased risk of developing carcinoma of the mouth, pharynx and esophagus. Carcinogens such as tar, benzopyrene, carbon monoxide and nicotine contribute to this risk. Early-stage oral cancer is often presented as painless, bright red lesions with granular or smooth surfaces. Smoking cessation improves survival rates and reduces the risk of recurrence [2].

## Periodontal and gingival tissues:

Smoking impairs immune response by reducing salivary immunoglobulin levels (IgA, IgG) and suppressing leukocyte activity. Nicotine disrupts the functioning of periodontal ligament cells and promotes pathogen colonization. Smokers exhibit increased pocket depth, alveolar bone loss and tooth mobility, which can lead to tooth loss. Periodontal disease also contributes to oral malodor. Acute Necrotizing Ulcerative Gingivitis (ANUG), marked by painful, bleeding, necrotic gingiva, is more prevalent among heavy smokers due to compromised humoral and vascular responses. Studies confirm smokers are more susceptible to ANUG due to immune dysfunction and poor oral hygiene [4].

## Implant stability in smokers:

Smoking negatively affects implant stability by interfering with wound healing and osseointegration. While primary stability depends on mechanical factors such as implant design and bone quality, secondary stability, which is essential for long-term success, is biologically driven and can be delayed in smokers. Evidence shows implant failure rates are nearly twice as high in smokers and up to four times higher in heavy smokers (>20 cigarettes/day). Though findings on stability are mixed, some studies suggest slower osseointegration and reduced secondary stability in smokers [5].

## Oral mucosa, dental plaque, halitosis and salivary changes:

Tobacco smoke causes epithelial alterations such as smoker's melanosis and stomatitis nicotina. The latter presents as keratotic nodules on the palate resembling a cobblestone pattern and are common among reverse smokers and pipe users due to thermal irritation. Halitosis is common among smokers due to volatile sulfur compounds and reduced salivary flow [6]. Smoking also accelerates plaque and calculus buildup, compromising oral hygiene and increasing periodontal risk. Smokers exhibit elevated salivary thiocyanate levels, useful as biomarkers for tobacco exposure. Reduced cystatin levels impair enzyme inhibition, weakening oral defenses. Smoking is also linked to increased dental caries due to higher microbial counts of Streptococcus Mutans and Lactobacillus [6].

## Determinants of tobacco use and oral disease:

Tobacco use, in both smoked and smokeless forms, remains a major modifiable risk factor for oral disease, including periodontitis, oral cancer, mucosal lesions; dental caries, delayed wound healing and implant failure. Persistently high tobacco consumption and low screening rates highlight the urgent need to expand tobacco cessation and oral cancer screening services, specifically among socioeconomically disadvantaged populations [7]. The determinants of tobacco use extend beyond individual behavior and are rooted in social, economic, cultural and environmental contexts [8]. The World Health Organization (WHO) defines the social determinants of health as "the circumstances in which people grow, live, work, age and the systems put in place to deal with illness" [9]. Political, social and economic inequities influence exposure, vulnerability and access to cessation support, thereby shaping patterns of tobacco use and oral health outcomes. Education and income are among the strongest predictors, with higher socioeconomic status associated with improved access to health education, while lower socioeconomic groups, often characterized by limited education, exhibit higher tobacco use and more severe oral disease [10]. These disadvantages are further compounded by intersecting factors such as race, ethnicity, cultural identity, LGBTQ+ status, mental health and substance use disorders [11, 12]. These populations frequently encounter targeted tobacco marketing, limited access to cessation programs and weaker policy enforcement, creating environments where tobacco use is normalized and cessation is difficult. Behavioral factors such as frequency, type and duration of use, as well as repeated risk behaviors such as alcohol consumption, poor diet and inadequate oral hygiene, further amplify oral disease risk [10, 13]. Second hand smoke exposure, particularly in children, increases caries incidence [13] while low motivation among adults perpetuates chronic exposure. Although cessation reduces future risk, prior tissue damage maintains elevated lifetime susceptibility [9], making primary prevention and avoidance of initiation the most effective strategies. Oral healthcare providers play a crucial role in supporting cessation, with provider knowledge, confidence, training and the frequency of dental visits influencing the delivery of behavioral interventions [7].

## Behavioral interventions in dental settings:

Dental professionals routinely observe the oral effects of tobacco, which creates natural openings to discuss smoking. Approaches such as Motivational Interviewing and the 5 R's help guide these brief conversations and offer supportive direction for patients who are considering quitting. The application of behavioral interventions within dental settings for smoking cessation has garnered considerable attention in recent research. Behavior change techniques (BCTs) are central to the success of these interventions. A comprehensive review by Moafa *et al.* analyzed behavior change techniques (BCTs) used by oral health professionals. The study found that interventions incorporating goal setting, readiness-to-quit assessment, self-efficacy support, action planning and personalized oral health feedback significantly improved cessation outcomes. These strategies yielded odds ratios ranging from 2 to 5.25, indicating strong effectiveness when correctly applied [14]. A recent Cochrane review by Holliday *et al.* showed that dental professional led interventions increase quit rates compared with usual care [15]. Evidence from Rasool *et al.* showed that dentist delivered counseling with personalized oral health feedback improved cessation among smokeless tobacco users [16]. Digital tools also show strong potential. Bricker *et al.* found that an ACT based smartphone program achieved higher abstinence than standard digital tools [17]. Long-term follow-up studies, such as those by Sujatha *et al.* demonstrate that moderate-intensity behavioral interventions in dental clinics can yield adequate quit rates over extended periods. Their randomized trial indicates that sustained behavioral support within dental settings can be effective in promoting long-term abstinence, reinforcing the value of ongoing behavioral counseling [18]. Despite these benefits, several barriers limit widespread implementation. Time pressures during consultations, limited training, low provider confidence and lack of financial reimbursement are frequently cited challenges [19]. Training initiatives that emphasize behavioral counseling methods have been shown to improve provider preparedness and increase the regularity of cessation support in practice [20]. Overall, incorporating behavioral approaches into routine dental care supported by organizational systems and ongoing clinician training can lead to better tobacco cessation outcomes. Given the heavy global burden of oral diseases caused by tobacco use, dental practices represent an underutilized but essential setting for advancing public health strategies in smoking cessation [19].

## Pharmacological approaches for smoking cessation:

Medications are a central element of modern smoking cessation strategies because they target withdrawal symptoms, cravings and nicotine's reinforcing effects. Research shows that pharmacotherapy nearly doubles the likelihood of sustained abstinence compared to minimal support. The most effective agents include partial agonists of nicotinic acetylcholine receptors (nAChRs), such as varenicline and cytisine, as well as combination nicotine replacement therapy (NRT). Bupropion and certain tricyclic antidepressants also help, though to a lesser extent [21].

## Nicotine Replacement Therapy (NRT):

Nicotine replacement therapy (NRT) aims to temporarily replace much of the nicotine from cigarettes to reduce motivation to smoke and nicotine withdrawal symptoms. This approach helps lower the urge to smoke and supports individuals in transitioning from active smoking to complete abstinence [21]. NRT options like patches, gum, lozenges, inhalers and sprays supply controlled nicotine doses that ease withdrawal without exposing users to the harmful byproducts of tobacco smoke. A Cochrane review of over 130 trials found all approved NRT products significantly improve quit rates compared with placebo or no treatment, with little difference between products when used properly [22]. Combining long-acting patches with fast- acting forms, such as gum or sprays, yields better outcomes and is recommended in clinical guidelines [23]. Tailoring doses to smoking intensity and advising short-acting NRT in anticipation of known triggers further enhances real-world effectiveness [24].

## Partial agonists:

## Varenicline and cytisine:

Varenicline selectively targets the α4β2 nAChR, easing withdrawal while blocking nicotine's rewarding effects. Evidence, including the large EAGLES trial, shows varenicline achieves higher quit rates than bupropion, NRT, or placebo without increasing serious psychiatric side effects even in individuals with mental illness [25. Nausea is the most common side effect, but it can be managed through gradual dose escalation, food intake, or dosage adjustments. Treatment typically lasts 12 weeks, extendable to 24 weeks for relapse prevention [25]. Cytisine, a plant-based nAChR partial agonist, is gaining renewed interest. A randomized trial showed higher short-term abstinence rates with cytisine than with NRT and most side effects were mild and gastrointestinal. Updated Cochrane reviews support cytisine as effective and cost-efficient, though varenicline typically achieves greater absolute effects [26].

## Antidepressants:

Bupropion SR, a norepinephrine-dopamine reuptake inhibitor with nicotinic antagonist activity, improves cessation rates over placebo and performs similarly to single-agent NRT, though less effectively than varenicline [27]. Common side effects include insomnia and dry mouth; seizure risk is rare but contraindicates use in individuals with seizure or eating disorders. Nortriptyline is effective but rarely used as a first-line due to anticholinergic effects and monitoring requirements [27].

## Optimizing treatment and safety:

Treatment should reflect dependence severity quit history, comorbidities, side effects, cost and patient preferences. First-line choices often include varenicline or a combination of NRT. Bupropion is an option for patients concerned about weight or depression, assuming no contraindications [22]. Combining medication with behavioral counseling yields the best results. Extending therapy beyond 12 weeks helps prevent relapse. Switching or combining therapies is preferred over abandoning pharmacologic support [24]. In pregnancy, behavioral support is first-line, with NRT considered if benefits outweigh risks. Both NRT and varenicline are safe in cardiovascular disease [28].

## Vaping and its oral health implications:

Electronic cigarettes, also known as e-cigs, vapes, vape pens, e-hookahs, tanks, or electronic nicotine delivery systems (ENDS), are smokeless alternatives to traditional tobacco products. The Centers for Disease Control and Prevention (CDC) reported that they are now the second most widely used tobacco product among adults. Approximately 3.7% of adults report using them [29]. The most common effects reported by the World Dental Federation (FDI) were mouth and throat irritation and periodontal damage [30]. Vaping can alter the oral microbiome by promoting the growth of harmful, cavity-causing bacteria, such as Streptococcus mutans, while reducing levels of beneficial species, such as Streptococcus sanguinis. This imbalance increases the likelihood of developing gum disease and tooth decay. Research also suggests that dental implant success rates are lower in people who vape compared to non-smokers and non-vapers. Additionally, concerns exist regarding the acidic nature of e-cigarette liquids, which may contribute to enamel erosion and increased tooth sensitivity. Dry mouth is another frequent issue among vapers, often leading to discomfort and challenges with eating, swallowing and speaking. A reduction in saliva flow compromises the mouth's natural defenses, raising the risk of cavities and gum disease. Persistent dryness also makes individuals more susceptible to bad breath and opportunistic infections, such as oral thrush [31]. Research has shown that individuals who use e-cigarettes are more susceptible to periodontal disease, experiencing tooth and bone loss, compared to non-users, though these effects occur at a slower rate than in traditional cigarette smokers. Two cohort studies found that e-cigarettes disrupt the oral microbiome, promoting the growth of opportunistic pathogens in a way that mirrors the effects of conventional tobacco products. Other studies have pointed to a heightened inflammatory response, with evidence showing that e-cigarette use increases levels of inflammatory mediators and periodontal cytokines involved in gum disease development. Additionally, exposure to e-cigarette vapor has been linked to toxic effects on oral cells, including DNA damage, cell death, reduced metabolic function and diminished cell viability and proliferation. Notably, many of these inflammatory and cytotoxic effects appear to occur regardless of nicotine levels, suggesting a role for flavoring compounds and particulate matter. However, chronic, long-term use of nicotine-containing e-cigarettes has explicitly been associated with severe periodontal disease and tooth loss [29]. Dentists are advised to counsel patients on the adverse effects of vape use on oral health and overall risks and to offer support for quitting in collaboration with the patient's GP or pharmacist. For individuals who smoke or vape, routine oral mucosal screenings should be carried out during every dental visit to help detect early signs of oral changes. Patients should also be encouraged to monitor their mouths for any abnormalities and seek dental advice if they notice anything unusual. Additionally, when healthcare professionals such as GPs counsel patients about smoking or vaping, they should take the opportunity to highlight potential oral health impacts and refer patients for a dental assessment when appropriate [31].

## Dental and community anti-smoking program:

Despite the significant impact of tobacco on oral health, cessation counseling remains underutilized in dental practice, partly due to limited professional training and referral pathways. Evidence suggests that structured interventions delivered in dental settings, when combined with community-based programs, can significantly enhance cessation outcomes [32]. Community initiatives expand the reach of these efforts by addressing socioeconomic and cultural determinants of tobacco use, while dental professionals provide direct, individualized support. The integration of both settings thus represents a complementary model for improving cessation success and reducing tobacco-related disease burden.

## Courage to Quit (CTQ):

The Courage to Quit (CTQ) program is a structured, community-based and evidence-based smoking cessation intervention developed by the Respiratory Health Association of Metropolitan Chicago. It combines psychoeducation, behavioral, cognitive and motivational strategies to support quitting, offered in either a 6-session full version or a 3-session condensed version [33]. It has demonstrated strong feasibility (75%) and high acceptability (95%) among racially diverse and underserved urban populations, with quit rates reaching 36% consistent with tobacco treatment guidelines [34]. Its cost-effectiveness and adaptability make it a pragmatic solution for addressing tobacco use. Predictors of success include readiness to quit and use of cessation medications [33].

## Oral Health 4 Life (OH4L):

The Oral Health 4 Life (OH4L) trial tested an oral health promotion program integrated into state tobacco quitlines. In a randomized study of 718 socioeconomically disadvantaged smokers, OH4L combined standard cessation counseling with oral health advice, resources and tools. While the program did not significantly increase dental care utilization, it showed modest, though not statistically significant, improvements in smoking cessation, especially at 2 months [35]. The study demonstrated that quitlines are a feasible platform for delivering additional public health interventions though cost and access barriers limited dental outcomes. OH4L was evaluated in the context of three state quitlines; however, interested states can assess the program's effectiveness and make an informed decision [36].

## Dental professional's 5a model:

Smoking cessation reduces risks and dentists are well-placed to help through the 5A (Ask, Advise, Assess, Assist, Arrange) model, counseling, pharmacological aids and even brief 3-minute interventions [36]. Most dentists ask and advise, but fewer assess readiness, assist, or arrange follow-ups due to limited training, time, resources and confidence. Patients generally welcome advice but lack awareness of oral health impacts and quitting resources. With stronger training and support, dental settings can play a decisive role in tobacco control. Strategies such as Ask-Advise-Connect could significantly help enhance patient outcomes. Studies show dental professionals follow the 5As model with decreasing involvement from Ask to Arrange.

[1] Ask: Up to 94% of dentists routinely ask about smoking and record it, though they rarely assess types of tobacco beyond cigarettes.

[2] Advice: Between 40-97% advise patients to quit and discuss health risks/benefits, though some groups (e.g., only 8% of Australian dental students) show very low rates.

[3] Assess: Only 19-40% of dentists assess patients' readiness to quit, except U.S. periodontists, where rates were much higher (74%).

[4] Assist: While 55-85% report helping patients quit in general, specific actions (quit plans, quit dates, educational materials, medications, nicotine replacement) were much less common, often below 40%.

[5] Arrange: Few arrange follow-up or referrals (1-47%), though around 59% re-evaluate tobacco use at later visits [37]. Dentists are strong in screening/advising but less consistent in deeper cessation support. Dental schools should include practical smoking cessation training, motivational interviewing and referral systems. Modifications like the Ask-Advise-Connect model show promise for improving patient enrollment by directly linking patients to quitline services, though its use in dentistry remains limited [37].

## Conclusion:

We show that both conventional cigarette smoking and vaping pose substantial threats to oral health, adversely affecting oral mucosal integrity, periodontal structures and the long-term success of dental implants. Sustained reductions in tobacco use and meaningful improvements in oral health outcomes continue to depend on coordinated, multidisciplinary strategies. Integrated cessation treatments, including behavioral counseling, community support, focusing on strategies like the 5A's model and nicotine replacement therapy, should be practiced reducing these harmful effects.
